# Experience in Evaluating AAL Solutions in Living Labs

**DOI:** 10.3390/s140407277

**Published:** 2014-04-23

**Authors:** Juan Bautista Montalvá Colomer, Dario Salvi, Maria Fernanda Cabrera-Umpierrez, Maria Teresa Arredondo, Patricia Abril, Viveca Jimenez-Mixco, Rebeca García-Betances, Alessio Fioravanti, Matteo Pastorino, Jorge Cancela, Alejandro Medrano

**Affiliations:** Life Supporting Technologies, Universidad Politécnica de Madrid, Avenida Complutense 30, Madrid 28040, Spain; E-Mails: dsalvi@lst.tfo.upm.es (D.S.); chiqui@lst.tfo.upm.es (M.F.C.-U.); mta@lst.tfo.upm.es (M.T.A.); pabril@lst.tfo.upm.es (P.A.); vjimenez@lst.tfo.upm.es (V.J.-M.); rgarcia@lst.tfo.upm.es (R.G.-B.); afioravanti@lst.tfo.upm.es (A.F.); mpastorino@lst.tfo.upm.es (M.P.); jcancela@lst.tfo.upm.es (J.C.); amedrano@lst.tfo.upm.es (A.M.)

**Keywords:** ambient assisted living, living lab, verification and validation, evaluation guidelines

## Abstract

Ambient assisted living (AAL) is a complex field, where different technologies are integrated to offer solutions for the benefit of different stakeholders. Several evaluation techniques are commonly applied that tackle specific aspects of AAL; however, holistic evaluation approaches are lacking when addressing the needs of both developers and end-users. Living labs have been often used as real-life test and experimentation environments for co-designing AAL technologies and validating them with relevant stakeholders. During the last five years, we have been evaluating AAL systems and services in the framework of various research projects. This paper presents the lessons learned in this experience and proposes a set of harmonized guidelines to conduct evaluations in living labs.

## 1. Introduction

The world's population is living longer as life expectancy continues to rise. It is estimated that the portion of the population aged over 60 years will grow to about two billion by the year 2050 [1]. Such growth generates an ever increasing number of older adults who are unable to live independently and who are in need of assistance because of age-related or disease-related conditions. This situation is concurrent with the increasing number of people with physical limitations due to a mixture of aging and sedentary lifestyle effects [2,3]. The accelerated population growth brings about escalating healthcare costs and rising caregiver's burdens. To respond to such predicaments, researchers have been working in recent years on the development of innovative technologies to provide independent and safe assisted living solutions, which could help mitigate the impending aging population challenges [4,5]. These assistive technologies, which are generally based on the concepts of ambient intelligence (AmI), are commonly referred to as ambient assisted living (AAL).

The “European Next Generation Ambient Assisted Living Innovation Alliance” defines AAL systems as “intelligent systems that will assist individuals for a better, healthier and safer life in the preferred living environment, and covers concepts, products and services that interlink and improve new technologies and the social environment” [6]. The main goal of AAL is to provide, with the help of technology, home-independent living assistance for older adults and individuals with chronic conditions and physical limitations, in such a way that they may carry out their daily activities independently and safely, to stay healthier, maintain a good quality of life and extend their lifespan, autonomy, self-confidence and mobility [2,6–8]. These assistive services are provided through an interactive environment based on ubiquitous sensing, individual-environment interaction, context awareness, learning information systems, interaction with users and their relatives, as well as with external care services [6,8]. Some of the more representative examples of services that could be provided through AAL are: medication management [9,10], emergency response [11], fall detection [12], video surveillance [13], monitoring the activities of daily living [6], issuing reminders [14] and communication with friends and family [15,16]. Many other kinds of useful interventions and services could be provided, as well, including dangerous situation alarms, mobility assistance, cognitive support, tele-health services, service and social robotics and home automation and domotics, to mention only a few among the wide panoply of possibilities [5,7,17–19]. All these assistive services may be deployed within different environments, as for example: at home, in the work place, in community public spaces, inside healthcare institutional settings, *etc.* [17,18].

AAL systems are designed using distributed software components, based on ambient intelligence technologies, such as context managers, profile and service managers, reasoners, user interfaces and security managers [8,20]. Such components have to integrate all kinds of electronic devices embedded in the environment that communicate among themselves. Typical devices are sensors, actuators, gateways, personal computers, smart-phones, home appliances, domotic devices, assistive robots, wearable devices and e-textile, *etc.* [4–6]. The incorporation of a large variety of hardware and software from different manufacturers and technologies into AAL systems turns them into complex systems, whose design process should be given special consideration. Given the complexity of setting up an AAL system, the ensuing technology and specific technical procedures to be used should require little or no effort from the end-users, in accordance with the so-called zero-effort technologies philosophy [21].

Some of the most important aspects that should be given special consideration when designing an AAL system are users' needs, adaptation and personalization, natural and multi-modal human computer interfaces, accessibility, usability and user acceptability, among others [5–7,22–24]. Sometimes, AAL systems do not satisfy the real needs of end-users, causing dissatisfaction and poor motivation to use the system. In most cases, this is caused by a wrong design that does not take into account the real needs and capabilities of end-users. This problem could be solved by studying the reality of end-users and by directly including them in the development process, from the initial concept through systems design and prototypes [6].

The design of cost-effective and truly successful AAL systems must contemplate the early detection of errors, to be able to apply fixes well in advance of undertaking any improvements, validations or user tests. Failures or misuse of AAL systems can lead to useless systems or even pose life threatening situations [4,22]. For this reason, technologies and simulation tools should be used in all design phases to carefully check and validate all system functionalities. Another source of complexity is the diversity of the final users of such systems: elderly, disabled people, healthcare professionals, friends and relatives, *etc.* Because of this variety of needs and points of view, a comprehensive system evaluation might result in an extremely complex, costly and time-consuming process or even be impossible to fully achieve [25]. A good methodology is essential to support the validation of AAL systems [23,25,26]; without a methodical evaluation process, it might be difficult for the designers and developers to create an adequate AAL system that fully addresses the users' needs. In this sense, computer-assisted and virtual reality tools, as well as smart environments, such as living labs, could be of significant assistance for prototyping, simulating and testing AAL systems, by allowing virtual and real users to interact in a realistic manner with the AAL environment.

A living lab can be defined as “an experimentation environment in which technology is given shape in real life contexts and in which (end) users are considered ‘co-producers’” [27]. Living labs can be seen under the perspective of both the technology, *i.e.*, the infrastructure that simulates realistic environments, where controlled experiments can be conducted (e.g., a home, a school) [28], and the methodology, *i.e.*, user involvement, requirements elicitation, experimentation *etc.* [29].

Few comprehensive methodologies that explicitly involve the use of living labs exist that are applied to ICT (Information and Communications Technologies) and, particularly, to AAL. One of the most comprehensive ones is defined in [30], where Ståhlbröst defines the FormIT methodology. From the lessons learned in the use of the living lab in three projects, Ståhlbröst then elaborates ten guiding principles for experimenting with living labs, namely: identify, inform, interact, iterate, involve, influence, inspire, illuminate, integrate and implement. The proposed principles guide researchers in understanding how data about user needs can be collected, generated and understood through a living lab way of user involvement processes. In [31], FormIT is extended, including the role of the living labs through the whole lifecycle of a product, from the identification of needs to design to evaluation. While FormIT is a relevant example, it is focused on users involvement and requirements elicitation and provides few insights about the technical and validation aspects that could be of interest for researchers, especially engineers. Other works, like in [32], are more focused on user experience evaluation. Their work includes technical aspects and an architecture for integrating objective and subjective measurements together with the context of use, but is centered on mobile applications and does not necessarily involve living labs as part of the infrastructure. As regards AAL in particular, some interesting experiences are shown in [33–36]. In these publications, living labs are used in AAL for testing technologies, discussing them with stakeholders, validating assumptions and extending collaborations. Even though these works are relevant for understanding the role of living labs in AAL, none of these present concrete suggestions and guidelines.

This article describes our experiences and discusses our learned lessons during the last five years from evaluating AAL systems and services in our Smart House Living Lab, within the framework of several AAL-related research projects. These projects embrace different aspects of AAL service provision from the perspective of different stakeholders. While most projects take the point of view of elderly users, people with disabilities and people with chronic diseases (PERSONA—PERceptive Spaces prOmoting iNdependent Aging, AMIVital, CogWatch, REMOTE—Remote health and social care for independent living of isolated elderly with chronic conditions, universAAL, ParKinect), others focus on the development process and the availability of tools adapted for the domain (VAALID—Accessibility and Usability Validation Framework for AAL Interaction Design Process, EvAAL, universAAL). Given the variety of users, different validation techniques have been explored, such as task performance, questionnaires, experts reviews, heuristics analysis, structured interviews, walkthroughs, *etc.*, which were used to test and validate different aspects of the proposed solutions, like usability, effectiveness, learnability, emotional response, satisfaction, attractiveness, workload, *etc.* Based on this experience, we propose a set of recommendations useful when conducting evaluations of AAL systems using the advantages of the technologies typically available within living labs.

The rest of the article is organized as follows: Section 2 describes the Smart House Living Lab, hosted at the Universidad Politécnica de Madrid, and how it has been used in several AAL-related research projects. Section 3 describes the lessons we have learned and gives suggestions for other researchers in the field, and finally, Section 4 derives some conclusions and ideas for future work.

## 2. Experience in Evaluating AAL

### 2.1. The Smart House Living Lab

The mission of the Smart House Living Lab is to help the research and development of ambient intelligence technologies and services to prevent, care and promote the health and wellness of people and support the social inclusion and the independent living of fragile and dependent groups, in all stages of the value chain: training, experimental research, technological development and technology transfer. The laboratory has been developed in the CIAMI (Experimental Centre of Ambient Intelligence Services and Applications) project under the Plan Avanza Program of the Spanish Ministry of Industry, Tourism and Commerce, and it is managed by the Life Supporting Technologies Group (http://www.lst.tfo.upm.es/) of the Universidad Politécnica de Madrid [37].

**Figure 1.**
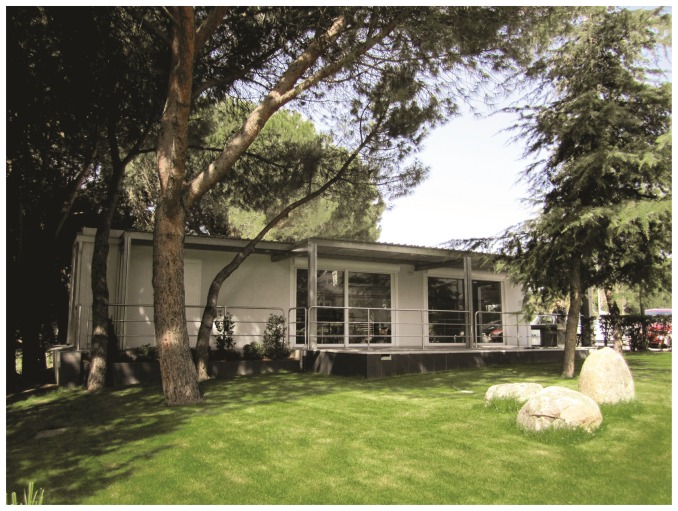
The Smart House Living Lab.

The Smart House Living Lab is a house (see Figure 1) created to be accessible by elderly and disabled users, equipped with the usual facilities of a conventional house and with added ICT devices (sensors and actuators) distributed extensively and hidden in ceilings and walls.

The Smart House Living Lab provides support to both developing new applications based on the massive use of technology distributed under the ambient intelligence paradigm and to testing and evaluating the quality of third-party applications and services that require a user-friendly environment with high connectivity and interoperability.

The main purposes that the Smart House Living Lab serve are the following:

- To facilitate the study of the needs of target users of applications and services: elderly people, people with disabilities, people with dependence on others and people who suffer chronic diseases.
- To help the development of technological solutions that satisfy specific needs in terms of health and social care at home for citizens (security, entertainment, social interaction, communications, information). Particular emphasis is given to the improvement of human-machine interaction for people with little computer literacy or physical or cognitive impairments.
- To intensively evaluate ambient intelligence applications and services. The lab can be exploited to assess both the technological aspects and user experience to make prototypes ready to be exploited and installed in real environments.

**Figure 2.**
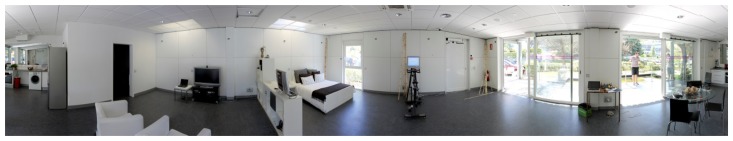
The overall view of the user area of the Smart House Living Lab.

**Figure 3.**
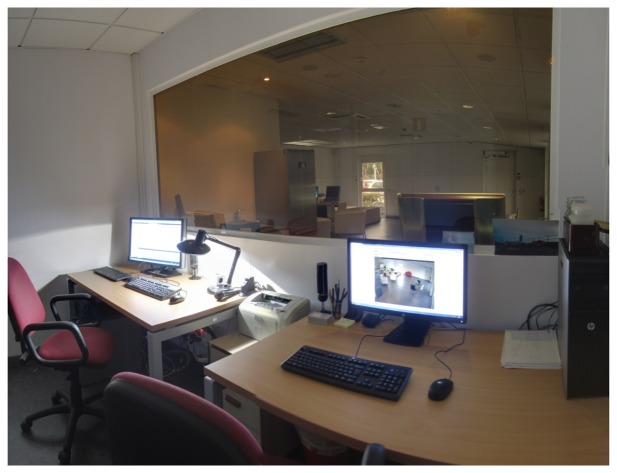
The overall view of the observation area of the Smart House Living Lab.

**Figure 4.**
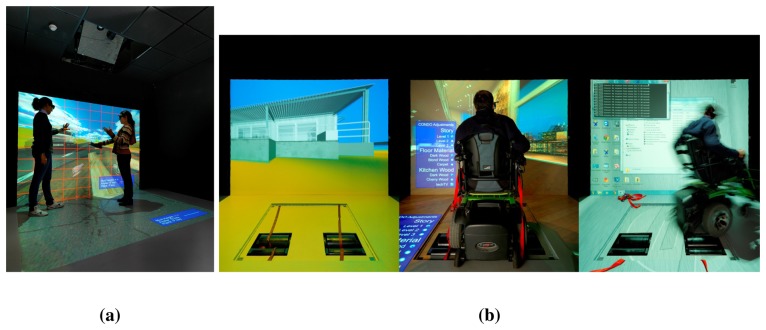
The virtual reality facilities of the Smart House Living Lab. (**a**) View of the virtual reality (VR) room; (**b**) VR wheelchair adaptation.

The Smart House Living Lab is divided into three areas:

1. User area: approximately 100 *m*^2^ equipped as a standard home with kitchen, bathroom, bedroom and living room, where the user can interact with the environment through natural speech, touch screens and hidden sensors (see Figure 2). The area is built as an open space, where also different scenarios, such as an office, can be simulated if needed.
2. The Observation room from which it is possible to have a complete view of the user space (excluded the bathroom) through a one-way mirror. The room holds the communication systems with high bandwidth Internet access, a server rack, networking facilities, like switches, cable patch panels and DIN (Deutsches Institut fur Normung) racks, all designed to be easily expandable in the future (see Figure 3). The availability of the observation room enables live control over the user centered experiments with techniques, like the Wizard of Oz, where subjects interact with a system that subjects believe to be autonomous, but which is actually being operated or partially operated by an unseen human being.
3. Virtual reality (VR) room used for studying both the user interaction with devices prior to their physical prototyping and for training in their use (see Figure 4a). It is also a key infrastructure for validating different scenarios and services in a simulated environment. The room is equipped with an *ad hoc* haptic wheelchair interface (see Figure 4b) to do experiments with disabled people.

**Figure 5.**
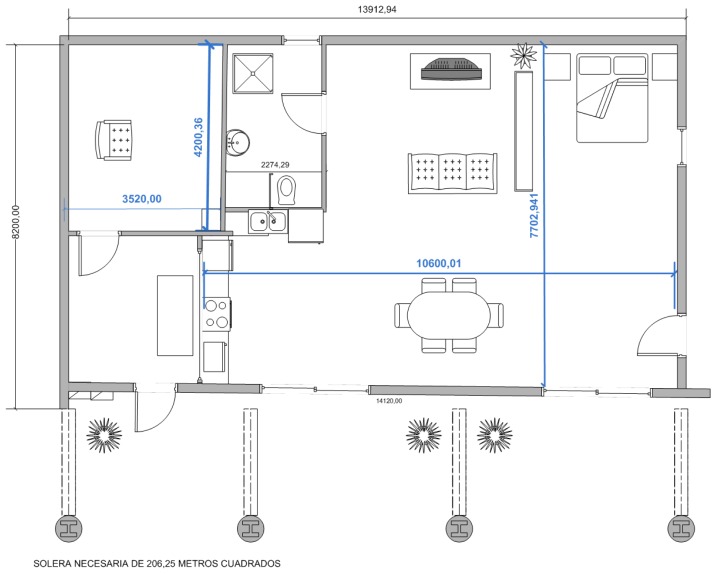
Map of the Smart House Living Lab.

The embedded technology in the living lab consists of more than 50 sensors and actuators connected to a KNX (Konnex) bus, such as: motion sensors, magnetic contacts in windows and doors, light sensors, blinds and doors actuators, smoke sensors, flood sensors, light dimmer actuators, fire sensors and heat and air conditioning systems. Apart from the sensors and actuators, the living lab has a set of distributed video cameras, loud speakers in all the rooms and microphones that enable communication from the user area to the observation room and *vice versa.* Cameras and microphones also allow the recording of users' activities and the analysis of their behavior *a posteriori*. Figure 5 shows a map of the living lab.

As part of the infrastructure, the living lab provides an experimental software platform, where open buses allow the easy integration of sensors and actuators. On top of this platform, an application interface is provided to developers for the rapid development of new services and to facilitate semantic interoperability among existing services.

### 2.2. Experience in Research Projects

In this section, the experience acquired in evaluating AAL systems in research projects is described. Table 1 shows a summary of the mentioned projects.

#### 2.2.1. PERSONA

PERSONA was a European co-funded project, which aimed at advancing the paradigm of ambient intelligence through the harmonization of AAL technologies and concepts for the development of sustainable and affordable solutions for the social inclusion and independent living of senior citizens, integrated into a common semantic framework [38,39]. The main outcome of the project was an open platform that provides a broad range of AAL services to support daily life activities, which was demonstrated and tested with a set of services in real-life conditions. The PERSONA platform embedded different sensor technologies (e.g., smart and intelligent textiles and embedded sensors, in-door location tracking system, ZigBee technology) and different means of user-system interaction, such as TV and LCD flat panels, voice recognition, touch, sound and lights.

The developed AAL services were designed to address the elderly users' needs for social integration and inclusion, support in daily life activities and security and safety when staying home or in mobility. All these services were evaluated by real users in simulated and real-life conditions in pilots in three countries. In the case of the Spanish pilot, evaluations were performed simultaneously in living labs in Madrid and Valencia. In the case of Madrid, two AAL services were selected for the evaluation: the Risk Manager, which detected other risky situations, such as falls or a stove turned on without any kitchenware on it; and the Nutritional Advisor, which helped the user cook and follow healthy nutritional habits.

A three-step evaluation methodology was followed for the assessment of user requirements and the acceptability and usability of the selected services: (1) preparation; (2) planning; and (3) user evaluation. During the first phase, the selected services were installed in a tactile PC in the kitchen area of the living lab. In the second phase, an evaluation plan was elaborated considering methodological and practical aspects. The plan included a set of sequential tasks that the users had to perform during the evaluation and a Likert-type scale questionnaire to measure the usefulness of the services, easiness of use, effectiveness, learnability and emotional response.

In the last phase, a total of five users were involved in the validation of the services. As an introduction to participants, an evaluator described the services and the evaluation plan. Then, the evaluator guided participants through the performance of the planned tasks and took notes of all the relevant participants' comments. After the task performance, participants were invited to fill out the questionnaire.

The benefits of conducting these tests in the living lab were appreciated. Participants felt confident about testing the services in familiar conditions, which also positively affected the acceptability of the services. Participants appeared to be talkative, proactive and critical; therefore, the results of the evaluation were rich and interesting.

Additionally, the presence of the control room in the living lab allowed the participation of more than one evaluator in a non-intrusive way. Nonetheless, several technical problems occurred during the evaluations. Sensors were not well integrated with the PERSONA platform, and in order to evaluate the Risk Manager service, evaluators decided to follow a Wizard of Oz approach to perform the planned tasks. The participants did not realize there were technical problems, and the evaluation of the service was not endangered.

#### 2.2.2. AMIVital

AMIVital was a Spanish funded project aimed at providing a platform to ease the development of applications and business models for ambient assisted living. The platform creates a digital personal environment for health and well-being innovative services to be consumed by the elderly, the disabled and people with chronic diseases [40,41] using bio-medical, personal and ambient sensors. Three general types of services were offered within the AMIVital platform: infrastructure services (logs, databases, communication service, *etc.*), general purpose services (calendar, health measurements, *etc.*) and functional services that integrate infrastructure and general purpose services to create complex processes and functionalities, such as tele-care, tele-medicine, tele-monitoring, remote consultation, medication assistant, *etc.* Furthermore, a context management service was included in order to integrate and analyze the information gathered with all the different devices used. As a proof of concept, the final AMIVital platform was deployed in the Smart House Living Lab, to prove the platform could be installed and used in real-life conditions.

The experiment consisted of two phases. The first phase had the aim of testing, from a technical point of view, the AMIVital installation in a real home. During two weeks, researchers performed a battery of integration tests to assess the reliability and the stability of the whole system, the communication of the different modules and possible integration problems. After technical tests, a set of tasks were defined for end-users to perform, including both short and long duration tasks. The objective of these tasks was to let users try all the implemented functionalities of AMIVital during a normal daily routine and to measure the acceptability and the attractiveness of the platform. These user tests allowed for validating the platform functionalities and service performances [42].

The results of these experiments were used as a proof of concept of the AMIVital project. The set up was recycled as a demonstrator to showcase the results of AMIVital. Technical testing in real environmental conditions, along with the logging and debugging features the living lab provided, were very useful to solve the interoperability issues of the platform. Nonetheless, the study was executed in a very small scale; other labs conducted experiments with more users.

#### 2.2.3. CogWatch

CogWatch is a European co-funded project aimed at providing a personal healthcare system (PHS) able to improve the standard rehabilitation process of patients suffering from apraxia and action disorganization syndrome (AADS) [43–45]. The developed system is characterized as affordable, customizable and capable of delivering continuous cognitive rehabilitation at home. It analyses the data coming from intelligent objects (daily use objects with embedded sensors, Kinect cameras and smart watches), giving multi-modal feedback through speakers, vibro-tactile actuators and visual displays that guide patients to accomplish the task; making them aware of errors and how to react.

The CogWatch system was tested by a large group of users without cognitive impairments during approximately one month in order to evaluate the technical and usability aspects of the integrated prototype. These tests constituted a preliminary evaluation before the extensive clinical validation with real patients, to assure the compliance with the requirements. The Smart House Living Lab offered a large, comfortable space in which to invite users. The Cogwatch system was installed and integrated with the living lab.

**Figure 6.**
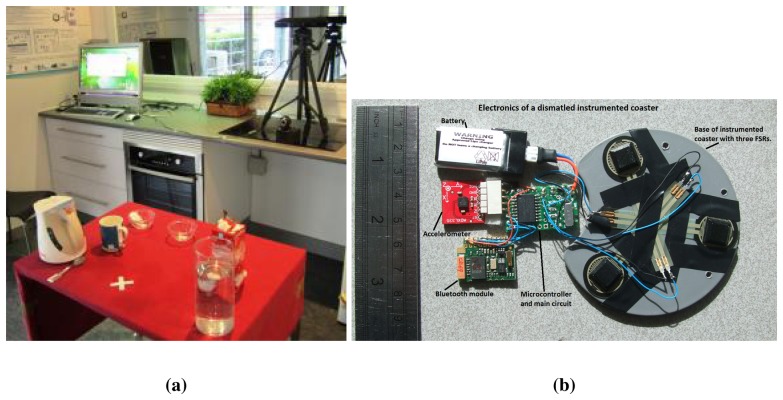
The test settings of the CogWatch system. (**a**) The CogWatch test workspace; (**b**) An example of the CogWatch smart tool.

A specific scenario was set up for technical and usability tests. Particularly, the test consisted of instructing the users to prepare tea, while the system was monitoring and guiding the user. The setting was as follows: a kitchen table was prepared with a set of smart tools able to track the user activity (as shown in Figure 6b), *i.e.*, a tea mug, an electric kettle and a milk jug with embedded pressure sensors and accelerometers, for motion tracking, connected through wireless Bluetooth communication. Other standard objects were also placed: A tea spoon, a sugar bowl, a box containing tea bags, a box to throw out the used tea bags and a water jug. A touch screen PC was placed close to the participant to provide users with the system's feedback. Two types of message cues were produced: text messages and audio messages, though audio messages were preferred (80% of the participants) to textual information (20%) to let the users concentrate on their task, to avoid looking at the monitor and reading the text cues. Figure 6a shows an overall view of the user work-space.

An *ad hoc* protocol was conceived of to test the system in terms of usability. The main performance indicators were: the user task performance rate and time, the usability questionnaires, the logging of spoken comments and direct observations. A short description of CogWatch was given to the invited participants, then the purpose of the tests and each task to be realized were explained. Particular emphasis, during the system outcomes analysis, was devoted to identifying the main technical bugs of the system and its less intuitive parts in order to improve prototype performance in terms of robustness and user acceptability.

Users were asked to complete a pre-defined set of tasks consisting in preparing three types of tea using the provided tools. Their behavior, during the interaction with the system, was recorded, and their comments were copied out. Two evaluators were present during the process to collect feedback and to check that the system was working correctly. In the case of technical problems, the test was not repeated.

Once the tasks were completed, participants were asked to fill out a questionnaire, in order to identify usability issues in the existing prototype. The questionnaire was created using three main standards of the usability heuristics approach: system usability scale (SUS) [46], to evaluate the subjective usability of the system; NASA Task Load Index [47], to evaluate the subjective workload assessments; and AttrakDiff [48,49], to evaluate the subjective attractiveness of the system.

At the end of each session, all the information collected by sensorized tools, notes and questionnaires were stored, and the settings were prepared for the following test. In total, twenty-five people participated to the tests, divided into two age ranges: less than sixty years old (13) and more than sixty year old (12). At the end of the tests, a total of 78 sessions were recorded. Among these, 67 were correctly completed, while 10 sessions were aborted, due to system errors. So far, the evaluation of the CogWatch prototype brought useful feedback in terms of system stability and user friendliness. This pilot served mainly to prepare the system for the final assessment with real patients, a task that is currently under development.

#### 2.2.4. VAALID

VAALID was a European research project that developed advanced computer aid engineering tools to allow AAL developers to optimize and make the whole process of user interaction design more efficient; and to validate usability and accessibility at all development stages, following a user-centered design (UCD) process [24]. The usage of VAALID tools makes it feasible, both economically and technically, to design AAL solutions that have the potential of being accepted by most users; since their needs are taken into account proactively during the development phases. To fulfill this vision, the VAALID project developed an integrated development environment (IDE) for the designers of AAL solutions. The VAALID IDE focuses on the design of the interaction between an older person (called the “beneficiary” in the VAALID project context) and the AAL solution. Furthermore, VAALID IDE provides the designer with tools to evaluate the accessibility and usability aspects of such interactions. Using VAALID 3D simulation environment, developers and designers are able to define and test AAL scenarios in virtual environments (VEs), and users can experience new interaction concepts and techno-elements, interactively.

One of the interaction interfaces developed in the context of the project was integrated and evaluated in the Smart House Living Lab. It consisted of an Android-based portable terminal for user interaction in a 3D virtual environment [50] The interaction was based on a wireless connection between a computer running a 3D environment and an Android application running on the smartphone. The 3D virtual environment was rendered in the living lab smart TV and in the virtual reality room, and the user could navigate along through the three-axis accelerometer provided by the smartphone, using the trackball and the touchscreen. Vibration feedback was provided when there were collisions with the elements of the VE. The validation phase in the smart house involved 10 developers with a relevant experience in the conception and design of AAL solutions. The validation was structured in four phases: (1) developers were trained to use the Android-based interaction interface; (2) participants were handed a structured, closed-ended evaluation questionnaire, which constituted a preliminary instrument to collect personal information (*i.e.*, expertise, previous experience with virtual environments, previous experience with AAL, *etc.*); (3) developers were asked to perform five different tasks involving several interactions with objects of a virtual environment using the smart-phone. During the performance of the tasks, evaluators collected quantitative data related to completion times, errors, difficulties, *etc*; the evaluators also took notes of the participants' comments; and (4) a final usability questionnaire was filled out by each participant at the end of the session.

These experiments provided interesting results and insights and showed promising potential for this type of interaction. The living lab infrastructure was key for the data collection, as it was possible to perform video and audio recording, as well as for the availability of the VR infrastructure.

#### 2.2.5. REMOTE

REMOTE was a European research project within the ambient assisted living joint program concerned with the needs of older people and individuals with chronic conditions, especially those at risk of living in geographical or social isolation. It aimed at providing support for individuals to have an independent life at home with the aid of ambient intelligence and tele-healthcare systems [51].

The project developed an open reference architecture and platform driven by ontologies enabling interoperability, seamless connectivity and content sharing among different applications and services for independent living. Users were equipped with off-the-shelf medical devices managed by mainstream computing units, such as laptops or smart-phones, that could connect to vital sign monitors. The recorded data was transmitted to the medical center and could be accessed on-line by groups of authorized medical professionals. Users had remote access to various other services that completed the support of independent living; services like personalized guidance on nutrition, physical or mental exercise, socialization and scheduled activities, as well as for control of their home devices.

REMOTE was validated with different types of target users (in total, 496 individuals) through its pilots in seven countries with relatively high percentages of population aged 65+ years. Validation in the REMOTE project was divided into validation at a technical level and the user's perspective. Regarding the environment, tests were carried out in three different scenarios: laboratory conditions (technical validation by developers), pilot conditions (user validation by experts) and real conditions (user validation by real users).

The Smart House Living lab was a key infrastructure for the evaluation of the REMOTE system using this methodology and, specifically, for the evaluation of the “Environmental Control at Home” service, which was specifically designed and implemented for the lab. This service enables users to control the domotic appliances of the smart house from their PC according to the following scenarios:

- Control a room: The user selects one of the house's rooms and controls the appliances, e.g., switching the lights on/off, opening/closing the blind, opening/closing the door, *etc.*
- Activate a scene: Different situations (e.g., wake up scene, go out scene) can be activated, so that all the appliances related to the scene are automatically controlled according to pre-defined settings.
- Common controls: Additional items can be controlled, *i.e.*, temperature, air conditioning and heating.
- Check events history: The user can check the history of actions that have been performed in the smart house.

The evaluation process was initiated with a technical validation by developers to identify bugs or performance problems. This evaluation process revealed it to be critical to increase the reliability of the system before starting the deployment in real homes.

In addition to the technical evaluations, the first user validation process started with expert-based evaluation techniques, specifically through heuristic analysis, aimed at examining whether an application is easy to learn and usable [52], and expert walkthroughs aimed at examining whether the graphical user interface is compliant with recognized usability principles [53]. The advantage of these techniques is that they are inexpensive, can be done at the very early stages of the design process, even on non-working prototypes, and allow for identifying and correcting as many usability and accessibility problems in the design as possible before any serious development is started. During this evaluation phase, the usability experts, located at the Smart House Living Lab, went through the application/system prototype, interacted with its various functions and interfaces in order to examine whether it was easy to use and to learn and identified potential areas that could cause confusion or errors to its intended audience. The assessed prototype (implemented with the Pencil (http://pencil.evolus.vn/) prototyping tool) simulated the interaction paths of the designed service. When the expert interacted with the different elements, the test facilitator (an engineer of the REMOTE team) simulated the actual behavior of the domotic appliances by directly activating them (e.g., switching on the kitchen light when the expert pressed the corresponding button on the GUI). Running this evaluation in the smart house proved to critically enhance the experience, inspiring experts to provide rich feedback on the tool. Results were provided to the application developers as aggregated reports, compiling all the comments gathered during the tests.

After improvements were produced in the service prototype, the second evaluation phase was held with real users. Even though real user tests can be done at any stage of the product development lifecycle, this phase started when a working prototype of the applications was already available. This time, six potential users (*i.e.*, older people) were invited to the smart house to assess the usability of the REMOTE platform and, in particular, the “Environmental Control” service. The evaluation methodology was based mainly on the task success rate, direct observation and logging of comments and questionnaires. At the beginning of each session, moderators gave an overview of the REMOTE project, informed participants about the purpose of the test, made a brief description of the application that they were going to evaluate and then let participants start exploring it. Users were asked to accomplish some pre-defined tasks, with a total duration of one hour maximum. The think aloud method [54] was used during the evaluations to capture the users' thoughts and opinions while using the service, while moderators wrote down the comments. As a quantitative and objective method, the number of times the user successfully completed a task was counted to calculate the user success rate [55]. Finally, participants were asked to fill out the system usability scale (SUS) questionnaire.

The evaluation plan was executed: the integration of a remote platform into the smart home environment significantly enriched the user experience of the participants, and valuable feedback was collected that resulted in many bug fixes and usability improvements. The methodology followed proved to be successful and was highly recommended by the developers and evaluators for use also in future evaluations.

#### 2.2.6. UniversAAL

One of the problems related to the adoption of AAL in real settings is the maturity of the prototypes developed in the many AAL research projects. Currently, AAL is pushed mainly by technology, meaning that it is not always well designed for its final users and does not always cover real needs [6]. universAAL was a European project that aimed to reduce barriers to the adoption and to promote the development and widespread uptake of innovative AAL solutions. The project's goal was to create the reference design and reference development platform for the ambient assisted living domain. The project benefited end-users (*i.e.*, elderly people and people with disabilities, their carers and family members) by making new solutions affordable, simple to configure and easy to personalize and deploy. It benefited also solution providers by making it easier and cheaper to create innovative new AAL services or adapt existing ones, by composing existing components, services and external systems [20]. universAAL was built with state-of-the-art technologies: The chosen programming language was Java, with OSGi as the container; and for distributing services, it implemented a set of “buses” built on top of JGroups with the support of a semantic framework based on RDF (Resource Description Framework) that allowed specifying the semantic of every piece of interchanged information. A set of standard sensors protocols were embedded into the platform: KNX, ZWave and Zigbee for domotics and IEEE X73 for medical devices. The integration of devices was simplified by a proper hardware abstraction layer, and the integration of external services (e.g., web services) was also made possible by an adaptation layer that makes full use of the semantic framework.

Given the high level of ambition of the project, an evaluation framework for the verification and validation of the results was put in place [25]. The methodology was necessary to guide the evaluations in the wide spectrum of AAL stakeholders, including manufacturers, researchers, service providers, authorities and health organization, and of quality characteristics, like usefulness, robustness, ease of use, reliability, *etc.* The universAAL evaluation framework was based on two pillars: A framework for creating theories in software engineering [56] and the standard ISO 25000 for SW (Software) quality assessment [57,58]. The theory framework was used to formalize the main objectives of the evaluation, the so-called research questions (RQs). An RQ is formulated to address whether a certain quality characteristic of a given artifact satisfies a need of a particular user. The artifact identifies the “what” to evaluate, the user the “whom with” and the quality characteristic and the need the “how” (an example of RQ is “Does the usability of the service satisfy the need of a simplified interaction of elderly users?”). The selection of the needs, actors and artifacts was based on the analysis of literature sources, and the project's objectives and quality characteristics were derived from the ISO 25000. The ISO standard inspired also the steps to be taken for each evaluation. As the first step, the evaluation requirements are identified as research questions; then, the specific artifact, quality characteristics, users and needs are selected for an assessment. In order to design the evaluation, specific metrics and tools are selected for the assessment, and a plan is created considering both theoretical and practical aspects. When the design is completed, quality characteristics are measured, and final users are involved if needed. Finally, the measured values of the quality characteristics are compared to target values; the results of the evaluation are discussed, and recommendations about what and how to improve are provided.

During the project, different evaluations have taken place according to two types of users: technical and non-technical. Regarding technical users, the following methods were employed: on-line questionnaires with developers, focus groups, field tests, scenario-based architectural evaluations, expert reviews and software metrics. Concretely, the Smart House Living Lab was involved in field tests, where universAAL was installed and integrated with the local facilities.

Users were involved particularly to evaluate a set of AAL services developed in the project. A total of 47 users was invited to our living lab to perform evaluations, including 29 elderly users, nine informal caregivers (relatives), five formal caregivers (doctors and social workers), three service providers and one authority representative. The users' tests that most benefited from the Smart House Living Lab were those in which tasks included the interaction with devices integrated into the Smart House Living Lab that were easily connected to the universAAL platform. In these tests, the users followed the think aloud protocol, while the test conductor took notes from what he had observed in a diary. At the end of the session, users were asked to fill out a questionnaire, which was an adaptation of the standard SUS.

The Smart House Living Lab was an important element in the universAAL project, as it has served as one of the main test beds for the project. The conducted evaluations proved to be significant and helped in correcting relevant defects both from the technical and the end-user point of view. One of the problems we identified during the tests was the difficulty, for external developers, to test their SW artifacts on the lab remotely, without having direct access to the resources needed to monitor and debug.

#### 2.2.7. ParKinect

ParKinect is aimed at developing a home platform for rehabilitation and workout routines for Parkinson's disease (PD) patients [59]. PD is the second most common neuro-degenerative disorder after Alzheimer's disease, reaching 1% of the population over 60 years of age in industrialized countries [60]. In addition to multiple other effects, the impaired basal ganglia function in PD leads to alterations in gait and balance, which often restrict functional independence and are a major cause of morbidity and mortality among these patients [61–64]. The objective of ParKinect is gait rehabilitation through repeated gait exercises and, at the same time, to use entertainment platforms, such as the Nintendo Wii™or Microsoft®Kinect™, for the detection and quantification of the main motor symptoms of PD patients in order to build a personal profile of the disease for each of them. ParKinect proposes a set of exercises to improve the gait, balance and general body movement coordination in patients suffering from PD, monitoring PD patients and integration with the Smart House Living Lab to support PD patients in their daily activities.

**Figure 7.**
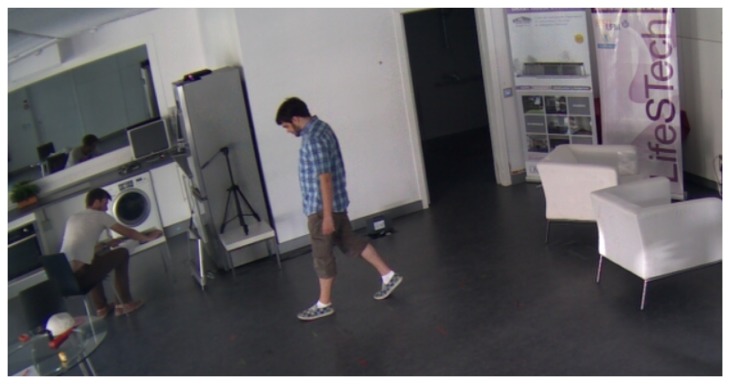
User testing ParKinect in the Smart House Living Lab.

For the initial evaluation phase of the project, we required a flexible environment to experiment with different set-ups and to explore diverse scenarios in a home atmosphere to perform this kind of tracking. The chosen sensor was the Microsoft Kinect, because of its capabilities and contained price. Using the skeleton information provided by the sensor, a finite state machine was built to automatically recognize and assess the motor performance of PD patients who followed visual and audio cues. The flexibility of the living lab allowed us to explore different heights and angles for the sensor position, as well as to validate the technical performance, *i.e.*, the ability to identify and track the walking of each subject, when they follow different paths in the setting. This last point is extremely important, since the accuracy of the measurements differs abruptly from one to another. The Kinect was placed at three different heights; for each height, all the users were asked to walk along different paths (each path was drawn with landmarks on the floor). Furthermore, three different auditory cues were played during the experiments (40 bpm, 80 bpm, 120 bpm). In this preliminary phase, 17 healthy subjects were involved in this experiment, with an average age of 28.52 ± 4.69 years old. Figure 7 shows one of the subjects performing a gait test.

For future work, the role of the living lab will become even more important. Besides involving patients in a controlled environment, where several variables can be analyzed in detail, the living lab allows us to extend our research outside the limitations of the traditional home locations. For example, it is possible to create a responsive environment according to the patients' needs, e.g., when a patient requires some help to start walking (freezer patients), the smart house could start playing auditory cues in the room where the user is and display visual cues in front of the patient.

#### 2.2.8. EvAAL

EvAAL (Evaluating AAL System Through Competitive Benchmarking) is an annual international competition, incubated by the AAL Open Association (AALOA(http://aaloa.org/)), which addresses the “grand” challenge of evaluation and comparison of AAL systems and platforms, with the final goal of assessing the autonomy, independent living and quality of life that AAL systems may grant to their end-users [65,66]. The main aim of the competition is the establishment of benchmarks and evaluation metrics to compare AAL solutions and to assess advances in the field. Given the complexity and heterogeneity of AAL systems, EvAAL has been focusing on the last three editions in the building blocks that compose complete AAL solutions. Particularly, the following topics have been considered: Indoor localization, activity recognition and companion robotics. The competition is held in realistic environments, living labs, where competitors can install their systems and test them according to a well-defined benchmark. For each kind of technology, different benchmarks are prepared, but usually considering similar aspects, like accuracy, availability of data, installation complexity, easiness of integration and user acceptance. During the competitions, data sets are collected and then made publicly available for researchers, and at the end of the event, a workshop is organized every year to nominate the winning teams, deliver prizes and to discuss the results of the competition and the involved technologies.

The Smart House Living Lab was involved in the execution of two editions, hosting the competition for indoor localization. Each team was required to present a localization system to cover the user area of the living lab, without limits to the number of devices or technologies. The assessed metrics for each competing system were:

- Accuracy, calculated as the 75th percentile of the error distance between the estimated localization sample and the reference position (ground-truth).
- Installation complexity, that is a function of the person-minutes of work needed to complete the installation of the competing system.
- Availability, measured as the ratio between the number of produced localization samples and the number of expected samples at a rate of two samples per second.
- User acceptance, which expresses how invasive the system is in the user's daily life, taking into account the presence of tags, the need to change batteries, how the solution is hidden in the environment, *etc.* It is implemented as a questionnaire to be answered by the competing team.
- Integrability with AAL systems, which evaluates the openness of the SW, the adoption of standards for both SW and HW (Hardware) and the replaceability of parts of the solution with other ones. It is implemented as a questionnaire to be answered by the competing team.

**Figure 8.**
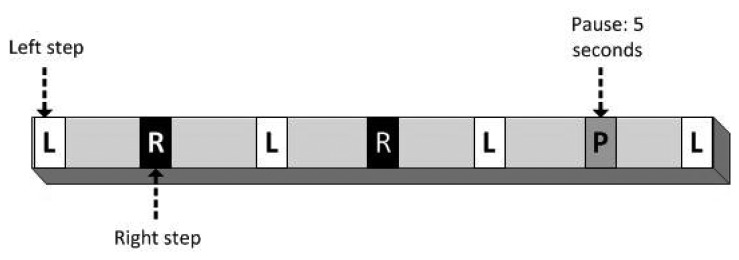
Segment used for signaling the path to the actor.

For each criterion, a weighted numerical score was then awarded and added to the overall score. The actual benchmark consisted of an actor walking on a pre-defined path at a predefined speed. The path was composed of straight segments, which were placed on the floor of the living lab, each segment comprising a set of marks that indicated left steps, right steps and pauses (see Figure 8).

Special software was in charge of guiding the actor, building the ground-truth and calculating evaluation metrics. The SW was built on top of the universAAL platform; thus, it used Java and OSGi, while the integration with the competitors' systems was realized with a simple ASCII-based *ad hoc* protocol on a TCP socket.

The SW was responsible for instructing the actor with sounds. Every second a sound was played to signal the actor to move one step forward or to enter or leave a pause. Thanks to this synchronization mechanism, and knowing in advance the position of all the segments, the SW was able to produce automatically the ground-truth, *i.e.*, the *a priori* position of the actor at every moment.

**Figure 9.**
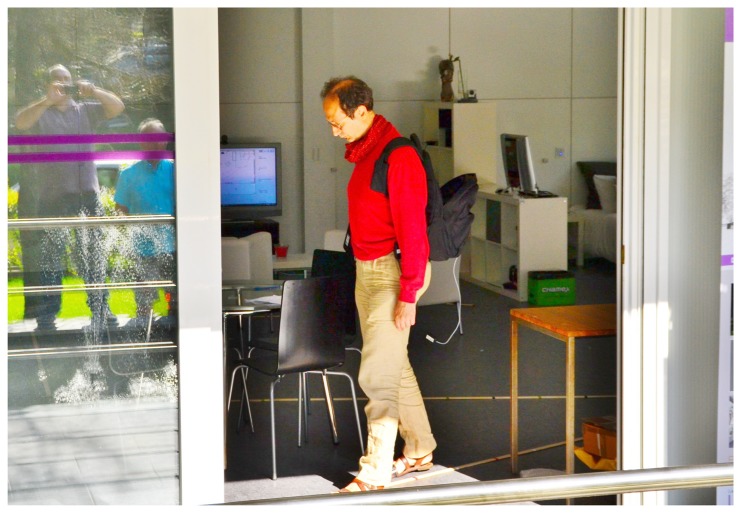
Actor moving on a path during a benchmark.

The benchmark was composed of the following steps:

1. In order to hide the actual path to the competitors and to not obstruct the team during the installation time, the sticks were removed from the floor before the competitors arrived.
2. When the competitor team reaches the hosting living lab, the local committee welcomed them and rules were explained.
3. The team started the deployment of the system, and the time for this operation was measured. In order to produce the meta-data for the datasets, the position of each device deployed in the living lab was also measured at the end.
4. The integration between the competitor's SW and the evaluation SW was checked.
5. The segments were placed on the floor and the benchmark started. During the benchmarks, only the actors were allowed inside the living lab (an example is shown in Figure 9). The evaluation SW produced the ground-truth and computed the scores relative to the accuracy and availability.
6. Different evaluation phases were performed: one related to the accuracy of the detection of “areas of interest”, one related to the measurement of punctual accuracy and one performed with a second actor in the room (called the “disturber”).
7. All benchmarks were repeated twice, and the best scores were taken of the two tries.
8. The competitor team was interviewed regarding user acceptance and integrability aspects.
9. The final score was computed. The competitors gave to the organizers the data sets produced by their systems; then, the team was allowed to witness the rest of the competition while giving access to the next participant.

In order to exploit the infrastructure of the living lab at its best, the competitors were given the possibility to receive events coming from electronic appliances of the lab, including all light switches, position sensors (PIRs) and a static bicycle. The actor was instructed to produce such events, and competitors were given the exact position of each device. Even though not all competitors took advantage of this possibility, those who did could correct their measurements in those moments when an event was available. The evaluation SW was based on the universAAL platform, thanks to which, the integration with the KNX infrastructure of the living lab was made easy, as the platform already provides the implementation of the protocols. universAAL also allowed the SW to be distributed among different devices. Concretely, we used one server for running the synchronization of the actor and the computation of the accuracy and availability metrics, two “slave” devices acting as user interfaces for the competitors, which showed a picture of the ground-truth path and the measured position, and the competitor's machine, which provided localization data and received events from the appliances.

The EvAAL competition has been so far successful, and an increasing number of participants have been competing each year. The quality of the technical solution, as well as the refinement of the evaluation criteria have been subject to iterative improvements. The complexity and the reliability of the SW proved to increase among the three versions of the competition. In the first competition, for example, a bug in the synchronization among time stamps of the computers prevented computing the accuracy correctly, and a second piece of SW had to be written to compensate for this problem at the end of all the benchmarks. The metrics chosen for the benchmarks proved to be robust to the wide variety of the competing technologies. Few adjustments were made, especially in the questionnaires for the assessment of user acceptance and integrability in order to make them completely objective and to distinguish between the current implementation and possible developments of the assessed system (the questionnaires are available at the EvAAL website (http://evaal.aaloa.org/)).

## 3. Lessons Learned and Recommendations

Based on our experience from the past five years, we have gathered, as a set of lessons learned and guidelines, all relevant information that could allow better planning and improve implementation of evaluations in AAL. This information should help develop new and better procedures for evaluating technologies, preventing or minimizing risks and support better bug-fixing and decision-making. We present the following lessons learned and recommendations according to four categories: technical aspects, methodological aspects, user aspects and ethical, legal and safety aspects.

### 3.1. Technical Aspects

During the above-mentioned projects, we have experienced some problems and limitations due to the way the living lab was set-up and how technologies were integrated. Here, we summarize our technical experience, and we provide suggestions.

#### 3.1.1. Device Failure

During some experiments, we had to fix problems that were related to the infrastructure that were hard to be detected. Apart from prototypes, even commercial devices can cause real headaches. As an example, we would like to cite a network switch that suddenly failed and started to flood the network and reducing bandwidth significantly. At the the beginning, we were blaming the prototypes, and it took two days of work to figure out where the source of the problem was. As a recommendation, though it may sound obvious, we encourage others to buy products of good quality and, most importantly, to install monitoring services for the hardware. Monitors should be installed at all levels of the infrastructure, from cabling and switching to access points, WiFi and domotic buses, like KNX.

#### 3.1.2. Quick Configurability

When the same infrastructure is shared by several projects at the same time, the easy configurability of the infrastructure is fundamental. In our experience, we had to repeatedly configure networks and devices for different scenarios, and particularly, networks may be subject to different requirements depending on the project. Our recommendation is to leave the possibility of separating networks physically, which also reduces the risk of having one prototype affecting the functioning of the rest of the infrastructure. Given the reduced dimensions of a living lab, it is feasible to separate network cables and converge them to a central point.

#### 3.1.3. Integration at Early Stages

Integrating heterogeneous systems is reckoned to be a difficult task. Interoperability and standards often solve most of the problems, though our experience shows that integration always hides problems, often related to nuances (e.g., protocols that do not follow the conventions properly, different operating systems presenting different behaviors). To address this issue, modern SW development methodologies, like extreme programming and continuous integration, suggest performing integration from the very beginning of a project. Regarding experimentation in living labs, we recommend following similar advice, *i.e.*, starting the integration of systems into the infrastructure from the very early prototypes.

#### 3.1.4. Separate Demonstrators from Systems under Development

Beyond working as a testbed, the living lab is also an exceptional place for running demonstrations for visitors and evaluators. In our experience, it is recommendable to separate a working prototype from the system under development or test. This separation may require setting up separate networks and separate computers and can be therefore costly; for this reason, agile configurability, again, is recommended when designing the lab infrastructure. When the artifact is purely software, virtual machines may help in setting up a completely closed system that could be switched on and off quickly and even replicated. In order to reduce the cross-influence of systems, it is always advisable to have quick solutions for switching on and off complete systems, including the server, clients, networks and other possible artifacts.

#### 3.1.5. Developers Can Be Users

Some projects we have worked on aimed at producing platforms that were meant to simplify the development of AAL services (e.g., PERSONA, VAALID or universAAL). In these projects, the actual target audience is the developers themselves. For developers, mostly the same techniques apply as for end-users, but with a different point of view. For instance, usability methods, like task performance, questionnaires and interviews, are perfectly applicable and can produce objective and interesting results. Task performance measurements were, in fact, created first for measuring the time of the execution of tasks in factories and are therefore oriented towards the working environment. One of the problems related with developers is that the results can be biased depending on if they are part of the project and on their experience and background. We therefore recommend using internal developers for making the first verifications and then identifying a good population (in terms of variety of expertise) of developers that are not working on the project. The limit of this latter approach is the cost, because the participants would be required to work, ideally for long periods of time.

#### 3.1.6. Log and Trace Problems

The value of evaluation is measured by the influence it brings to the development of the project and its contribution to correcting defects. In our experience, we have observed that sometimes, there is a lack of communication between the evaluators and the implementers of the solution. Tests are done, reports are produced, but the results are not taken into account. This can be for several reasons, including: a certain “inertia” of the developers to correct bugs (especially when those are related to non-functional aspects, like usability or security); poor technical knowledge or involvement of the evaluators, which leads to superficial and poorly usable feedback (e.g., “the system crashes” or “the user does not understand the interface”); lack of good tools that help to document and trace problems of the artifacts and identify the responsible for fixing errors. As a recommendation, we suggest to always have at least some technical developers involved during the tests, these being technical tests or user tests, and to adopt proper bug tracking tools that have to be shared among all the members of the project.

### 3.2. Methodological Aspects

In this section, we discuss the evaluation methodologies adopted in the above-mentioned projects. A summary of all the adopted methodologies is shown in Table 2, where it is possible to observe that a varied mix of methods has been used, including objective and subjective measurements. Even if objective measurements are usually preferable in engineering, given the nature of the experiments in living labs, it is expected that these measurements are not always achievable. Methods from social and behavioral sciences and case studies have to be adapted for the assessment of AAL systems, including those related to usability, user acceptance and health technology assessment. Following is a set of specific lessons learned, and recommendations are given.

#### 3.2.1. Identify Your Evaluation Needs

Before starting an evaluation, given the effort and resources needed to conduct it, it is very important to understand what is the relevant information that the project needs to retrieve from it. In our experience, it has happened that some evaluations were conducted with a superficial idea about what to assess, and some relevant information was therefore missed. Identifying the dimensions to be evaluated also helps to search for the best methods for the assessment and to optimize the process and reduce costs.

#### 3.2.2. Mix Evaluation Approaches

It is usually better to measure the same phenomenon with different means than having to trust one single measure. Whenever feasible, we recommend joining different evaluation approaches in order to be able to compare the results among the different assessment means and to better support the results. Applying different evaluation approaches also makes it more probable to have results comparable to other studies and, in the long term, to select the measurement mean that optimizes the effectiveness *versus* its cost.

#### 3.2.3. Prefer Standard Questionnaires, but Be Flexible

Use of a standard questionnaire is preferable for two reasons: it is validated by the scientific community, meaning that it measures what it claims to measure and it allows the comparing of different studies across each other. In our experience, we have always tried to adopt standard questionnaires, but we found that some well-known ones are sometimes too technical to be easily understood by elderly users (an example is the SUS). In this case, we suggest making an adaptation to the questions in a way that the meaning is kept, but the form is more easily understandable by the users.

### 3.3. User Aspects

Living labs are set-up especially for creating a more direct contact with users and stakeholders of AAL systems. This section is dedicated to lessons learned and suggestions about how to involve users, how to make them participate in evaluations and what to expect from them.

#### 3.3.1. Involve Users Gradually

Solutions should grow with the help of users, and users are (usually) not a cheap resource. It has happened, in our experience, that some evaluations were started before the system was sufficiently stable or usable. In these cases, though evaluations force one to quickly identify and fix problems, and thus produce advances, it is not advisable to “burn” the users with systems they cannot understand or that simply do not work. The results is that this will produce little confidence in the system and bias future assessments. The recommendation we can provide is to start with a reduced groups of users who are more confident with technology and who can “tolerate” errors more easily, and then, as the product quality increase, extend the user base.

#### 3.3.2. Establish Trust and Accommodate

Users usually participate in studies for curiosity or as a favor and very rarely for compensation, if any. Even in cases when compensation is foreseen, the amount is such that usually it is not the only motivation for joining a study. Therefore, it is necessary to accommodate and motivate users, and a friendly and sincere collaboration shall be established between them and the researchers. Users should be stimulated to tell stories about their situation, about their needs and the reasons why they would adopt or not a proposed system. In order to stimulate sincerity, users have to feel they are able to express their opinions freely, without hurting others' self-esteem. For this reason, we recommend letting them know that the developers of the solution they are testing are not present during the evaluations, even if this is not entirely true.

#### 3.3.3. Users Present Patterns

Every user is different, but usually, it is possible to identify some patterns in their behaviors and attitudes. In our experience, for instance, we have observed a general pattern that is almost always applicable: the skeptic and the enthusiast. The skeptic is a person who may understand the value of the technology, but considers himself as unable or unwilling to use it; the enthusiast, on the other hand, is a person who trusts technology and feels ready to adopt it. Between skeptics and enthusiasts, there are different intermediate profiles, but roughly, we have found that these two categories apply well, especially within elderly users. As a recommendation, we suggest not limiting the analysis of the results to the averages, but to try to cluster results and to identify possible classes of users.

#### 3.3.4. Let Users Learn

The first time a user operates a system, he or she can have problems in understanding the interface and the general behavior of the system. This is perfectly normal, especially considering AAL users who might be computer illiterate. We have observed that researchers during the experiments may be tempted to guide users to complete tasks in order to be more efficient and close the evaluations quickly. This kind of approach is counter-productive, because it hides usability problems and it makes users feel that they are not in control of the system or even confuse them more. We strongly suggest introducing the users to the system properly and gradually, with simple exercises, and to not interfere with their tasks. There are of course cases, because of evident usability problems, or with particular users, where the researcher should intervene to keep the timing reasonable. In those cases, though, all problems should be logged properly, and interferences should be made explicit during the analysis of the results.

#### 3.3.5. Perform Pre-Evaluations with Experts

Evaluations are always costly and have to be optimized. Involving users, setting up the environment and doing the tests is expensive and time consuming. One way to optimize this process is to do small-scale controlled tests without involving final users and exploit the experience of experts. In our experience, we have used experts' reviews for technical evaluations, but also for usability assessments, and we suggest it as a mean to detect and correct the most evident defects in a simple way.

#### 3.3.6. Understand Context

Evaluations always happen in a context, and people can have different reactions to different contexts. The behavior of the user has to be observed in the real world context; users must feel that they are in a natural and comfortable environment and perform the tests without pressure. Researchers must pay attention to all the interesting information that can be extracted from the users' context. Users should be motivated to tell their stories and give real-life examples, imagining how a certain technology would integrate into their homes and their daily lives. This way, researchers will be also able to improve the living lab experience, by simulating more realistic and convincing scenarios.

#### 3.3.7. Social and Cultural Differences Have to Be Considered

Users can differ extremely in their social, economic and cultural background. ¿From our experience, we have learned that is always better to have a good representation of different social classes and education levels. Especially important for ICT, more than scholarly education, is computer literacy. In the case of AAL, it is to be expected that the average user has little experience with computers, though it has to be emphasized that this situation is going to change rapidly for two reasons: first, because mobile technologies are starting to be popular also among the elderly; and secondly, because the elders of the following decade will most probably have used computers in their work.

#### 3.3.8. Cherish the Users' Experience

As for technical evaluations, users evaluations have to positively affect the development process. If developers are often sensible of technical issues, they can be less interested in user aspects and can ignore or underestimate those recommendations coming from user evaluations. The value of these evaluations is in the fact that they confirm if the technology covers real needs and in a proper way. Without this confirmation, technologies are destined to be kept in developers' labs. For this reason, we suggest producing comprehensive and clear reports after the evaluations are completed, with sensible suggestions for improvements, and we also recommend always inviting one or a few developers to take part in the user tests, so that they can learn and interiorize the way their users think and feel.

### 3.4. Data Collection and Analysis Aspects

In this section, we describe some of the common issues related to how data shall be collected and analyzed after the evaluations have been completed.

#### 3.4.1. Consider Failures

The system under test in a living lab is probably a prototype, and defects and failures are the norm in this case. Given that evaluations are costly, it is always necessary to take failure into account already while designing the evaluations. We have learned that a lot of time and effort can be spent on fixing problems that may arise during an evaluation and that these may compromise the evaluation itself. Making a decision when these events occur is not easy; users can be put on hold until the problems are fixed or tests can be repeated, but this will mean more time and resources will be spent. It is not easy to find a generally applicable rule for such cases, but as a recommendation, we suggest to be at least prepared, that is, to consider failures already when choosing users, setting up the tests and preparing the evaluation methodologies.

#### 3.4.2. If It Is Digital, It Is Better

We have experienced losing a considerable amount of time copying manually filled out forms to digital formats. As simple as it may sound, we recommend using digital means to collect all kinds of information, including logs, questionnaires, observations and notes. To help the process, there exists a plethora of programs and tools for all kinds of assessments; most of them are usually free or even open source (e.g., LimeSurvey for questionnaires).

#### 3.4.3. Exploit the Infrastructure

The main difference between a living lab and a normal home is that the lab comes equipped with all kinds of sensors and technologies. In some of the evaluations we have performed, the living lab was not strictly needed. A normal apartment or even an office would have been suitable for the purpose. There are two main reasons for involving the living lab in such experiments. First, the user experience is substantially enhanced. Even at early stages of development, when only non-functional mock-ups are available, performing evaluations in the living lab helps users to better understand the context and the potentiality of the product. Second, running evaluations in living labs can provide valuable information that otherwise could be impossible to retrieve thanks to the sensorized environment. The recommendation we give is to use all of the infrastructure, even though it is not strictly needed, therefore, to activate sensors, cameras and capture inputs and provide outputs during the assessments, because usually, the cost of doing so is reduced and the added value can be considerable.

#### 3.4.4. Share Datasets

Research is a process based on the accumulation and sharing of information. Currently, few initiatives are devoted to sharing datasets from living lab experiments, despite the fact that these data could be of immense value for researchers. Datasets can be used to train algorithms and to compare solutions in different settings. As a recommendation, we suggest build repositories of data, rich with information about the settings of the experiments and their purposes, and to make them available to the public, whenever possible.

#### 3.4.5. Use Standard Formats and Annotate

When analyzing data collected during tests, not to mention when sharing these datasets with other researchers, using well-known formats is fundamental for making it easy to parse and compute them. Examples of relevant standard formats for the field are: HL7 Clinical Document Architecture, SCP-ECG (Standard communications protocol for computer assisted electrocardiography) and DICOM (Digital Imaging and Communication in Medicine) for health related data; Geohash, GeoURL, indoorGML and ISO NP17438 for localization; Android's activities (http://developer.android.com/reference/com/google/android/gms/location/DetectedActivity.html) and the LIRIS HARL (Laboratoire d InfoRmatique en Image et Systemes d information—Human activities recognition and localization) dataset for activity recognition. In our experience, it has happened that unspecified formats and incompatibilities have slowed down the analysis of data, though most of the time, these problems are solved with proper communication among researchers. In those cases where standards do not exist for the kind of retrieved data, it is extremely important to describe the data formats and also annotate all the conditions under which these data were collected.

### 3.5. Ethical, Legal and Safety Aspects

A comprehensive study of ethical, legal and safety aspects is out of the scope of this paper and would require profound analysis. Nonetheless, we would like here to provide a few rules of thumb that can be generally applicable to tests in living labs.

#### 3.5.1. Safety First, Always

We understand that it is not possible that prototypes fulfil all security standards, but there are some basic principles that should be taken into account for any evaluation with users in a living lab. During a test, the individual might be exposed to physical damage, due to the erroneous design or behavior of the system. In order to be able to manage this risk, installations should be compliant with safety norms, especially if these are to be used for extended periods of time. In addition, any equipment should be pre-evaluated for personal safety. There must be a pre-defined plan to support the user in case he/she might be endangered by inappropriate system information.

#### 3.5.2. Informed Consent

Users must be able to voluntarily decide whether or not to participate in an evaluation. For that purpose, it is highly recommendable, and usually mandatory, to ask them to read and sign an informed consent document that includes all the significant aspects related to the objectives, methodologies, risks and benefits of the evaluation, as well as the data management procedures. Language and documentation must be written in a simple and easy to understand language. Alternative media (e.g., large print, audio tape, Braille) should be provided for users with special needs.

#### 3.5.3. Anonymity

Participants should be able to control the dissemination of the collected data during an evaluation. Researchers should not be allowed to circulate non-anonymous information. Therefore, only relevant attributes, *i.e.*, gender, age, *etc.*, should be retained. Personal information (e.g., identity, *etc.*) should be stored by one designated person (in a password protected file), only for the duration of the test of each user and then be deleted. Audio and video material should be kept confidential, as well, and preferably should not capture faces or other recognizable aspects.

#### 3.5.4. Ethical Committees

Any organization performing experimental work with human beings must have the authorization of the relevant ethics control committee. These committees must evaluate all the aspects of the evaluation to be performed and formally approve the experimental procedures. This is particularly important when the evaluation is related to scenarios that are related to medicine and life-supporting systems. Although, not always, AAL systems are critical for diagnosis or treatment, it must be ensured that all the basic principles of experimentations with human beings are respected.

## 4. Conclusions and Future Work

In the last five years, we gathered a great deal of experience evaluating AAL solutions in the Smart House Living Lab. As this paper shows, problems always arise when setting up experiments, even in highly controlled settings. ¿From methodological approaches to practicalities, it is preferable to be prepared to address problems before they appear.

We believe to have achieved the five key principles recommended by [30]: continuity, *i.e.*, cross-border collaboration based on trust; openness, *i.e.*, gathering many perspectives and involving users; realism, *i.e.*, generating results that are valid for real-life situations; empowerment of users: *i.e.*, engagement of users based on human needs; and spontaneity, *i.e.*, inspiring usage contributing to societal needs. All these aspects are fundamental for fostering good practices in experimentation with living labs, especially for the case of ambient assisted living.

The main lesson learned that we want to highlight is that living labs are a means for fostering user involvement in the development process and for increasing the maturity of a solution in terms of both technical reliability and usability before deployments in real homes, even at small scales. The value of the living lab relies on the fact that it helps in addressing real users' needs or, as Ponce de Leon *et al.* [67] said: “technology in itself is no longer valid; benefits and usefulness for people in their daily life must be proven before the technology or service can be said to be a success”.

As future work, we are aiming to create a set of standardized guidelines for AAL technology assessment and experimentation in living labs. These guidelines would include testing techniques for different classes of technologies, depending on their scope and the end-users. The final goal is to define standard procedures for certifying AAL technologies. Different aspects can be considered, also separately, including reliability, effectiveness, security, interoperability, extensibility, usability, *etc.*; nevertheless, the validity of this certification will depend on the acceptance that these guidelines will receive from both the research and the industrial stakeholders.

## Figures and Tables

**Table 1.** Summary of research projects. AAL, ambient assisted living.

**Project Name**	**Start/End Year**	**Field**	**Type of Users**	**Financing Body**
PERSONA	2007–2010	Social inclusion and independent living	Elderly users	EU
AMIVital	2007–2010	Applications and business models for AAL	Elderly and disabled users. People with chronic diseases	CENIT
CogWatch	2011–2014	Cognitive impairments	People with praxia	EU
VAALID	2008–2010	User interaction design, validation and accessibility	Developers with experience in AAL design	EU
REMOTE	2009–2012	Independent life at home	Older adults and individuals with chronic conditions	EU
universAAL	2010–2014	AAL solutions	Elderly users, people with disabilities, their carers and family members, developers and service providers	EU
ParKinect	2013–ongoing	Parkinson's disease	Parkinson's patients	—
EvAAL	2011–ongoing	AAL solutions	AAL companies and institutions	EU / volunteering

**Table 2.** Summary of the evaluation methodologies used in our projects. SUS, system usability scale.

**Project Name**		**Technical Tests**	**User Tests**
PERSONA	type of tests	integration tests	tasks performance, questionnaires (Likert scale)
	assessed dimensions	reliability	usefulness, ease of use, effectiveness, learnability, emotional response
AMIVital	type of tests	integration tests	tasks performance (short and long duration)
	assessed dimensions	reliability	acceptability, attractiveness
CogWatch	type of tests	integration tests	tasks performance, log of comments, questionnaires (SUS, NASA task load index, AttrakDiff)
	assessed dimensions	reliability, requirements compliance	satisfaction, attractiveness, workload, usability
VAALID	type of tests	integration tests	training of developers, questionnaire, interviews
	assessed dimensions	reliability	productivity, learnability, attractiveness
REMOTE	type of tests	integration tests, bug tracking	experts reviews, heuristics analysis, log of comments, think aloud, tasks performance, questionnaires (SUS)
	assessed dimensions	reliability, maturity	usability, attractiveness
universAAL	type of tests	questionnaires, focus groups, field tests, scenario based architectural evaluation, experts review, software metrics	questionnaires, structured interviews, walkthroughs, heuristics, tasks performance, think aloud
	assessed dimensions	reliability, maturity, suitability, performances, productivity	usability, suitability, attractiveness, effectiveness
ParKinect	type of tests	accuracy of the Kinect sensor for gait assessment	feasibility of the different paths and setups
	assessed dimensions	accuracy extracting gait features	succeed rate on each path and setup
EvAAL	type of tests	performances measurements, experts review, questionnaire	experts review, questionnaire
	assessed dimensions	accuracy, installation complexity, availability, integrability	user acceptance
